# Editorial: Development of selective carbon-11 radioligands for target-based PET molecular imaging in oncology, cardiology and neurology

**DOI:** 10.3389/fmed.2023.1137088

**Published:** 2023-01-23

**Authors:** Sridhar Goud Nerella, Manoj Kumar, Kiran Kumar Solingapuram Sai, Chander Singh Digwal

**Affiliations:** ^1^Department of Neuroimaging and Interventional Radiology (NI&IR), National Institute of Mental Health and Neurosciences (NIMHANS), Bengaluru, India; ^2^Department of Radiology, Wake Forest School of Medicine, Winston-Salem, NC, United States; ^3^Department of Medicinal Chemistry, National Institute of Pharmaceutical Education and Research, Hyderabad, India

**Keywords:** carbon-11, fluorine-18, molecular imaging (MI), positron emission tomography, radiotracers

This Research Topic gathered different contributions pertaining to the development of carbon-11-based radioligands for oncological, cardiovascular, and neurological positron emission tomography (PET) imaging applications. In addition, it has extended to cover fluorine-18 based radioligands and their imaging applications. Several collected articles have shed light on a variety of novel radiochemistry approaches, labeling procedures, and their role in drug development, and evaluation of radioligands in preclinical animal models.

Over the last three decades, the PET molecular imaging technique has emerged as a powerful tool for biomedical research, drug development process and medical diagnosis (1). It utilizes radiotracers that can detects changes at the molecular level or functional changes. These radiotracers are bioactive heterocycles labeled with a short-lived radionuclides, either carbon-11 (*t*_1/2_ = 20.4 min) or fluorine-18 (*t*_1/2_ = 109.8 min).

Carbon-11 is a versatile radionuclide because it can be inserted into a wide array of bioactive compounds to develop PET radioligands. Several secondary carbon-11 labeled synthons such as ^11^CH_3_OTf, ^11^CH_3_I, ^11^CO, ^11^CH_2_O, ^11^COCl, ^11^HCN, ^11^CS_2_ etc. can be generated using cyclotron produced [^11^C]CO_2_ or [^11^C]CH_4_ (2), thus which can be inserted into different heterocyclic systems. Fluorine-18 is also a widely used radionuclide for PET radiotracer development due to its longer half-life of 110 min., providing an option to transport the radiotracers to non-cyclotron centered medical or research facilities. In addition, long run radiochemical synthesis, quality control studies, and whole-body PET scans at later hours can be performed (3). Carbon-11 (non-carrier added) is produced in high molar activities (GBq/μmol), whereas the fluorine-18 (carrier added) is produced in low molar activities due to non-radioactive contaminations. Therefore, carbon-11 molar activities are mostly higher than fluorine-18 and makes it an attractive target for *in vivo* quantification of low-density target proteins.

The first article of this Research Topic (Nerella et al.) explains the importance of PET imaging technique for drug development. The fundamentals and principles of radiochemistry and PET imaging were discussed clearly with several examples. PET advances the drug discovery and development process by providing information about new target engagement, proof-of-mechanism, and proof-of-concept. Pharmacokinetic profiles and pharmacodynamic actions of new drug candidates provided by their corresponding radiotracers. The chemistry of radiolabeling with both carbon-11 and fluorine-18 was elaborated using both established and novel procedures. Additionally, several novel oncological and neurological biomarker-based radiotracers were discussed. Detailed discussion on the section of recently published patents for carbon-11 and fluorine-18 labeled radiotracers supported their systematic study. Overall, this review was intended to be a useful resource for chemists and biologist entering the molecular imaging research.

The [^11^C]MPC-6827 has been developed for imaging microtubules in Cocaine Use Disorder (CUD) by Damuka et al.. Authors have reported preliminary results of a potential imaging biomarker i.e., microtubules in CUD using the brain penetrant radiotracer, [^11^C]MPC-6827, in an established rodent model of cocaine self-administration. Both *in vivo* micro-PET/CT imaging and *ex vivo* biodistribution results showed lower (~35 ± 3%) brain uptake of [^11^C]MPC-6827 in cocaine self-administered rat brains compared to the control rats. *In vitro* autoradiography also suggested the low radioactive uptake in cocaine rats compared to the control ones. This study suggests that [^11^C]MPC-6827 uptake decreases in cocaine rats and that it may selectively bind to destabilized tubulin units in the brain. Overall, these findings may provide better understanding the biological underpinnings of CUD to develop new treatment strategies.

With an aim to compare the amyloid deposition at the lobar cerebral microbleed (CMB) sites in three different conditions namely cerebral amyloid angiopathy (CAA), Alzheimer's disease (AD), and cognitively normal healthy controls (NC), Chang et al. have conducted a pilot study using ^11^C-pittsburgh compound B (^11^C-PiB). Nine CAA, 15 AD patients, and 15 NC subjects participated in this pilot study. The standardized uptake value ratios (SUVRs) were measured at the CMB sites through PET/MR examination using ^11^C-PiB radiotracer, and cortical PiB distributions were quantitatively evaluated. Lobar CMBs were detected in all the CAA patients, eight of the 15 AD patients (53.3%), and four of the 15 NC subjects (26.7%), respectively. The SUVR values showed higher PiB deposition at CMB sites in CAA patients compared with AD patients and NC subjects. These findings indicated the CMBs occur preferentially at loci with concentrated amyloid; therefore, it may improve CAA diagnostic accuracy by combining both lobar CMBs and regional cortical amyloid deposition without histopathologic confirmation.

Jiang et al. reported a case study to find the characteristics of Prostate lymphoma (PL) that may be concurrently presented with prostate adenocarcinoma (PAC). A 32-year-old adult suffered from severe dysuria with normal prostate-specific antigen (PSA) levels. Initial pelvic MRI confirmed a mass in the prostate as well as multiple enlarged lymph nodes in the bilateral inguinal area, and the diagnosis of PAC was considered, but the serum PSA was normal. Hence, ^18^F-PSMA PET/CT was performed to find the characteristics of the lesion and to further guide the biopsy. However, no abnormal PSMA uptake was seen in the lesion of the prostate and lymph nodes of the pelvic cavity and bilateral inguinal area. These lesions were confirmed with increased glucose metabolism using ^18^F-FDG PET/CT followed by a prostate biopsy. The histopathologic examination confirmed the PL, and the patient subsequently received systemic chemotherapy and radiotherapy. The symptoms and the lesions completely disappeared after radiotherapy. The clinical symptoms of PL are atypical, and PL and PAC may be concurrently presented. High FDG-uptake and low PSMA uptake (by lymphoma) in the prostate was seen, which is opposite to the PAC. Hence PSMA PET/CT combined with FDG PET/CT could non-invasively identify the characteristics of PL.

An *in vivo* pig model for testing novel PET radioligands targeting cerebral protein aggregates was reported by Raval et al. A pig model was established with cerebral injections of α-synuclein based fibrils or brain homogenate from postmortem human Alzheimer's disease (AD) or dementia with Lewy body (DLB) brain tissue into the pig's brain. The model was validated with the amyloid-β radioligand [^11^C]PiB, which has higher affinity for β-sheet structures in aggregates. PET scans were performed post-injection of [^11^C]PiB, and protein expression was quantified using logan graphical analysis using the simplified reference tissue model 2 (SRTM2) with occipital cortex as the reference region. Post PET scans, the brains were retrieved to confirm successful injection of α-synuclein based fibrils or AD brain homogenates using autoradiography and immunohistochemistry. [^11^C]PiB showed four times higher brain uptake in AD-homogenate-injected regions and two times higher uptake in regions injected with α-synuclein-preformed-fibrils compared to saline, suggested that it is an excellent translational model for assessment of novel radioligands to image aggregated α-synuclein in a neurodegenerative brain.

Neuroinflammation is mainly associated with microglia activation and is strongly correlated with the translocator protein (TSPO) expression on the outer membrane of microglial mitochondria. Expression of TSPO in neuroinflammation can be assessed using [^18^F]GE-180 radiotracer. Routinely used 60–90 min time window post-radiotracer injection has not been suited for post-stroke neuroinflammation assessment. Therefore, Zatcepin et al. have compared semi-quantitative estimates obtained from late time frames to quantitative estimates of a full 0–90 min dynamic scan in a mouse model of photothrombotic stroke (PT). For the 60–90 min time window, excellent linear correlation was observed between SUVR and DVR. The extrapolated DVRs of the validation cohort did not show significant difference in the DVRs of the analysis group. Therefore, a comprehensive quantification of a dynamic scan can be replaced by a late 60–90 min post-injection of [^18^F]GE-180 PET scan to assess microglial activation in the PT mouse model.

Tsao et al. reported dual tracer PET/CT as an imaging probe to assess the *de novo* lipogenesis activity in the preclinical models of hepatocellular carcinoma (HCC). The dual tracers included^11^C-acetate and ^18^F-FDG. The results showed lipogenesis activity in various HCC cells with high uptake of ^11^C-acetate and low uptake of ^18^F-FDG, but there was no correlation between these two radiotracers uptake due to different mechanism (^11^C-acetate high uptake in lipogenic metabolism and ^18^F-FDG high uptake in glucose metabolism in HCC). Apart from providing information on the lipogenic activity, it also predicts the lipogenesis-targeted therapy response. For example, the HepG2 xenografts showed high uptake of ^11^C-acetate and low uptake of ^18^F-FDG indicating high lipogenic activity and that responded well with orlistat (a lipase inhibitor) treatment, whereas SkHep1 xenografts showed low uptake of ^11^C-acetate but high uptake of ^18^F-FDG indicating lower lipogenic activity, and high glycolysis, thus which show poor response to orlistat. Hence, this dual tracer PET/CT imaging can not only reveal status of lipogenesis and glycolysis but also predict the treatment response of HCC to lipogenesis-targeted therapy.

## Author contributions

SN: conceptualization, writing the original draft, review, and editing. MK, KS, and CD: writing, reviewing, and editing. All authors approved the submitted version.
